# Selective Gold Recovery from Homogenous Aqueous Solutions Containing Gold and Platinum Ions by Aromatic Amino Acid-Containing Peptides

**DOI:** 10.3390/ijms21145060

**Published:** 2020-07-17

**Authors:** Kin-ya Tomizaki, Takuya Okamoto, Tatsuki Tonoda, Takahito Imai, Masahiro Asano

**Affiliations:** 1 Department of Materials Chemistry, Ryukoku University, Seta, Otsu 520-2194, Japan; t16m053takuya@gmail.com (T.O.); tatsuki.tonoda.21@gmail.com (T.T.); imai@rins.ryukoku.ac.jp (T.I.); 2 Innovative Materials and Processing Research Center, Ryukoku University, Seta, Otsu 520-2194, Japan; 3 Course of Environmental Ecological Engineering, Ryukoku University, Seta, Otsu 520-2194, Japan

**Keywords:** peptide, self-assembly, noble metal recovery, gold, platinum

## Abstract

There is increasing interest in the development of noble metal separation/recovery processes, especially for applications to “urban mining”. Common separation/recovery processes for noble metals use a solvent (liquid-liquid) extraction technique in hydrometallurgy. However, these processes are time-consuming and not environmentally friendly, because they use organic solvents for sequential metal ion extractions. Electrowinning is an alternative approach for selective metal precipitation that involves controlling the redox potentials of electrodes but requires specialized equipment and generates hydrogen as a byproduct at the cathode surface under dilute conditions. In the present study, we investigated selective gold recovery from a homogenous aqueous solution containing a mixture of dilute HAuCl_4_ and H_2_PtCl_6_ (5.0 × 10^−5^ M each) and aromatic amino acid-containing peptides (2.0 × 10^−4^ M each). Gold selectivity was determined by analyzing the compositions of the solids and supernatants obtained from the reaction mixtures. A much higher gold selectivity (gold/platinum (Au/Pt) atomic ratio = 7.5) was obtained using an anthracene-containing peptide compared to peptides containing one or two naphthalene ring(s). Our proposed approach is applicable to the sequential separation of several noble metal ions, such as Au, palladium (Pd), Pt, iridium (Ir) and rhodium (Rh), and simply requires developing aromatics suitable for each noble metal of interest.

## 1. Introduction

Processes for noble metal separation/recovery are of increasing interest due to their applicability to sustainable chemistry. “Urban mining” is the recovery of metallic elements from industrial waste such as discarded electronic devices and may be more efficient than traditional mining, because electronic waste contains larger amounts of metallic elements relative to ores [1]. Common noble metal separation/recovery processes utilize a solvent (liquid-liquid) extraction technique in hydrometallurgy, in which gold (Au) is first extracted with a solvating organic extractant from an aqueous phase to an organic phase, and then palladium (Pd), platinum (Pt), iridium (Ir) and rhodium (Rh) are sequentially extracted with appropriate solvating organic extractants [2,3,4]. This process is well-established but suffers from bottlenecks and is time-consuming and not environmentally friendly because of the need for organic solvents to generate biphasic reaction systems for sequential metal ion extractions. Electrowinning is another method for noble metal separation/recovery by selective metal precipitation and involves controlling the redox potentials of electrodes. However, it requires specialized equipment and generates hydrogen as a byproduct at the cathode surface under dilute conditions. Furthermore, electronic waste generally contains dilute noble metal ions together with metal ion contaminants [5]. New noble metal recovery processes should meet the following challenging criteria: (i) high metal element selectivity, (ii) one-pot operation, (iii) easy separation and (iv) a monophasic reaction in (homogenous) aqueous solution under dilute conditions (i.e., a sustainable chemical process).

Gold nanocrystals have unique physical, chemical and biocompatible properties applicable for catalysts, sensing/imaging systems, photonic/plasmonic devices, drug delivery and photothermal therapy [6,7,8,9,10,11,12,13,14]. The properties of gold nanocrystals are strongly dependent upon their size and shape [15], and thus, there has been much effort to control the morphology of gold nanocrystals for the reduction of metal ions with aqueous solutions of reducing agents such as citric acid, ascorbic acid and NaBH_4_ [16,17].

**Figure 1:**
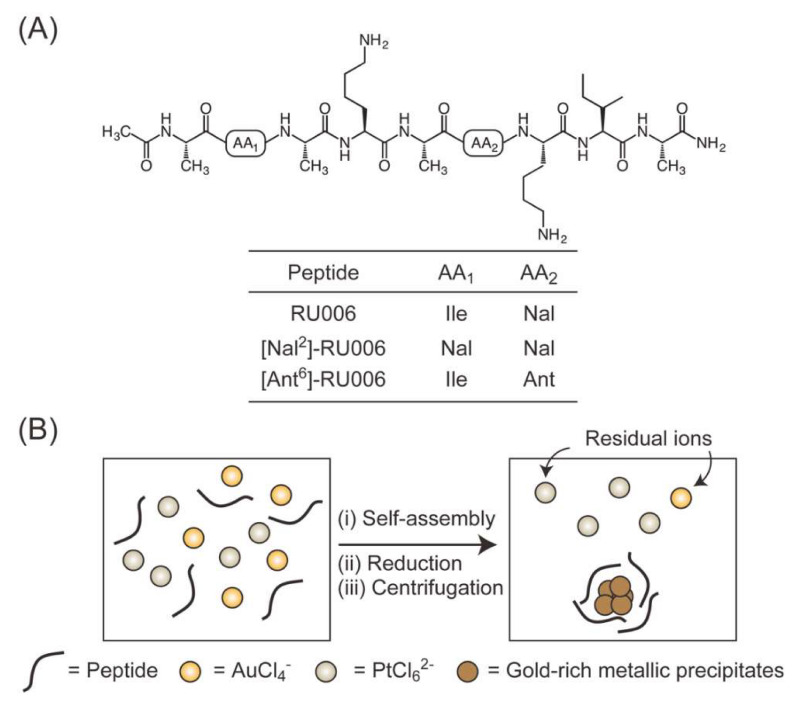
Amino acid sequences of the amphiphilic nonapeptides. (**A**) The original nonapeptide RU006 and (NaI^2^)-RU006 and (Ant^6^)-RU006. (**B**) Illustrative representation of selective gold recovery from a homogeneous aqueous solution containing gold and platinum ions by peptides. Abbreviations: NaI = L-2-naphthylalanine and Ant = L-2-anthrylalanine.

We previously designed and synthesized a β-sheet-forming nonapeptide (RU006: Ac-AIAKAXKIA-NH_2_, X = L-2-naphthylalanine, NaI) and fabricated gold nanoribbons 50–100-nm-wide, several nanometers high and with lengths of less than a micrometer by mixing RU006 and HAuCl_4_ in water (Figure 1A) [18,19,20]. This peptide-based gold nanoribbon synthetic strategy did not involve the addition of reducing agents required by traditional methods, because the naphthalene ring in the peptide acts as an electron source. We proposed the following mechanism for RU006-directed gold nanoribbon formation: (i) RU006 encapsulates and concentrates AuCl_4_^−^ in the interior cavities of peptide network architectures through electrostatic interaction with a cationic Lys side chain during peptide self-assembly stabilized by hydrogen bonds, hydrophobic interaction and/or π-π stacking among naphthalene rings; (ii) electrons are transferred from the naphthalene ring to Au(III) and (iii) gold crystals slowly grow to form gold nanoribbons along the template network architecture. The replacement of NaI (oxidation peak potential at 1.50 V) in RU006 with the stronger reducing aromatic ring anthracene (oxidation potential at 1.05 V) to provide (Ant^6^)-RU006 (X = L-2-anthrylalanine, Ant) altered the morphology of the gold nanocrystals from ribbons to spheres [18,19]. We determined that two and four electrons were transferred during the oxidation of each molecule of naphthalene and anthracene, respectively [19,21], suggesting that the difference in gold nanocrystal morphology using RU006 and (Ant^6^)-RU006 could be due to differences in the oxidation potential and the number of electrons transferred from the aromatic ring to Au^III^. Thus, the rate of reduction of metal ions by aromatics is likely important for controlling the morphology of metallic nanocrystals and may affect the selectively for noble metals in a metallic mixture. If so, then metallic elements of interest might be recovered selectively from a homogenous aqueous solution (no organic solvent) by the self-assembly of aromatic amino acid-containing peptides, rapidly enriching metal ions under dilute conditions (Figure 1B). Precipitates from such reaction mixtures must be sufficiently dense to separate by simple centrifugation, meeting the criteria for sustainable, green and noble metal recovery.

In this study, we investigated the selective gold recovery from a homogenous aqueous solution containing a mixture of dilute gold and platinum ions (each 5.0 × 10^−5^ M) by mixing the aromatic amino acid-containing peptides RU006 and (Ant^6^)-RU006. For comparison, we also tested (NaI^2^)-RU006, in which the isoleucine (Ile) residue at the 2nd position of RU006 is replaced by a NaI residue to provide a peptide with two naphthalene rings (four electrons per molecule) with the same oxidation potential as RU006 (Figure 1A). The selectivity of the peptide-based method for gold was examined by energy-dispersive X-ray spectroscopy-scanning electron microscopy (EDS-SEM) of precipitates formed by the reaction mixtures and collected by centrifugation to determine their metal element contents. Inductively coupled plasma-optical emission spectroscopy (ICP-OES) of the supernatants was used to determine the residual metal ion concentrations. We also determined the organic/inorganic compositions of the precipitates by thermal gravimetric analysis (TGA) to understand the utility of the peptidyl molecules in the present noble metal recovery system. Finally, we compared the selectivity of the peptide-based method for gold with methods using common reducing agents such as NaBH_4_ and ascorbic acid.

## 2. Experimental Section

### 2.1. General

All solvents and reagents, unless otherwise noted, were purchased from Wako Pure Chemical Industries (Osaka, Japan) and used as received. Fmoc-amino acid derivatives and reagents for peptide synthesis were purchased from Watanabe Chemical Industries (Hiroshima, Japan). Acetonitrile (HPLC grade) was purchased from Nacalai Tesque Inc. (Kyoto, Japan) and used for HPLC analysis and peptide purification. Ultrapure water was purchased from Wako Pure Chemical Industries (Osaka, Japan).

### 2.2. Preparation of Known Peptides

RU006 and (Ant^6^)-RU006 were synthesized according to the literature [18,19].

### 2.3. Peptide Synthesis and Storage

The peptides RU006, (NaI^2^)-RU006 and (Ant^6^)-RU006 were synthesized by Fmoc chemistry on Rink amide resin with 2-(1*H*-benzotriazole-1-yl)-1,1,3,3-tetramethyluronium hexafluorophosphate (HBTU) and 1-hydroxybenzotriazole monohydrate (HOBt) as the coupling reagents [22]. The amino acid side chain of lysine (Lys) was protected with *t*-butyloxycarbonyl (Boc). Final deprotection and cleavage of the resin-bound peptides was performed by treating with trifluoroacetic acid (TFA)/thioanisole/*m*-cresol (90/7.5/2.5, *v*/*v*/*v*) at room temperature for 60 min. The reaction mixtures were concentrated, then precipitated with cold diethyl ether to provide the crude peptides. The crude peptides were washed with cold diethyl ether three times, dried in vacuo and purified on a Hitachi LaChrom Elite HPLC System (Tokyo, Japan) using Cosmosil 5C_18_-AR-II packed columns (4.6 × 150 mm and 10 × 250 mm, Nacalai Tesque, Kyoto, Japan) with a linear gradient of acetonitrile/0.1% TFA at a flow rate of 1.0 mL min^−1^ for analysis and 3.0 mL min^−1^ for semipreparative purification. The purified peptides were lyophilized and characterized by matrix-assisted laser desorption/ionization time-of-flight mass spectrometry (MALDI-TOFMS; Shimadzu AXIMA-CFR Plus, Kyoto, Japan).

Peptide stock solutions were prepared by dissolving each purified peptide powder in 2,2,2-trifluoroethanol (TFE) to prevent self-assembly during storage. The concentrations of the RU006 and (NaI^2^)-RU006 stock solutions were determined by absorption spectroscopy (Shimadzu UV-3100 spectrophotometer; Kyoto, Japan) using an extinction coefficient of 5500 M^−1^ cm^−1^ at 276 nm for a NaI residue in aqueous solution containing 1% TFE (*v*/*v*) [23]. The concentration of the stock solution of (Ant^6^)-RU006 was determined by absorption spectroscopy using an extinction coefficient of 5300 M^−1^ cm^−1^ at 377 nm for an Ant residue in a methanol solution containing 1% TFE (*v*/*v*). The peptide stock solutions were stored at −20 °C until use.

### 2.4. Attenuated Total Reflectance Fourier-Transform Infrared Spectroscopy (ATR-FTIR)

The (NaI^2^)-RU006 films were prepared by sonication (Kaijo Sono Cleaner CA-44800, Tokyo, Japan) of a peptide aqueous solution ((peptide) = 1.0 × 10^−3^ M in water) at 50 °C for 2 min, followed by incubation at 40 °C for 1 day. ATR-FTIR spectra were acquired on a Shimadzu IR Prestige-21 FTIR spectrophotometer equipped with a Smiths Detection DuraSample IR II Diamond (Hertfordshire, United Kingdom).

### 2.5. Reduction of Noble Metal Ions with Peptides

Each peptide stock solution in TFE was transferred to a microtube, dried with a N_2_ gas stream and then dried in vacuo for 1 h. Water was added to the microtube to give a peptide concentration of 4.0 × 10^−4^ M, and the mixture was sonicated at 40 °C for 2 min to break up the peptide aggregates. Equivolume mixtures of peptide solution and aqueous solution containing noble metal ions (1.0 × 10^−4^ M) or a mixture of noble metal ions (each 1.0 × 10^−4^ M) were incubated in a dry block heater (As One EB-303, Osaka, Japan) at 40 °C.

### 2.6. Measurement of Absorption Spectra of Reaction Mixtures

Each reaction mixture was transferred from the microtube to a 1.0-cm-path-length quartz cell at specified time points, and UV-Vis absorption spectra were recorded on a spectrophotometer (Shimadzu UV-3100 spectrophotometer, Kyoto, Japan).

### 2.7. Field Emission-Scanning Electron Microscopy (FE-SEM) and Energy-Dispersive X-ray Spectroscopy-Scanning Electron Microscopy (EDS-SEM)

Each reaction mixture was centrifuged, and the supernatant was removed. The resulting precipitate was diluted with a small volume of water and dispersed. Droplets of the suspension were applied to a transmission electron microscopy (TEM) grid (Cu 200 mesh covered with a collodion membrane; Nisshin EM, Tokyo, Japan) for 1 min and dried with a filter paper. The SEM samples were dried in vacuo before measurement. FE-SEM images were acquired on a JIB-4601F (JEOL, Tokyo, Japan), and EDS analysis of the SEM samples was conducted on a JSM-6010LA (JEOL, Tokyo, Japan). Au/Pt atomic ratios for the precipitates were calculated by comparison of the intensities of X-ray signals characteristic for Au and Pt at three points randomly chosen within a sample.

### 2.8. Transmission Electron Microscopy (TEM)

TEM samples were prepared in a manner similar to that for FE-SEM and EDS-SEM, and measurements were conducted on a JEM-2100 (JEOL, Tokyo, Japan) at an accelerating voltage of 200 kV.

### 2.9. Inductively Coupled Plasma-Optical Emission Spectroscopy (ICP-OES)

The supernatants obtained by centrifugation of the reaction mixtures were analyzed on an Optima 5300DV (Perkin Elmer, Yokohama, Japan).

### 2.10. Thermal Gravimetric Analysis (TGA) of the Precipitates

The reaction mixture was centrifuged, and the supernatant was removed. The precipitate was diluted with a small volume of methanol and dispersed again. The methanol suspension was transferred to a platinum pan and dried. The TGA sample was mounted on a Thermo Plus TG8120 (Rigaku, Tokyo, Japan), and its weight was determined to be 0.365 mg at the starting temperature (40 °C). The temperature was increased linearly at 5.0 K min^−1^ from 40 to 400 °C

## 3. Results and Discussion

### 3.1. Design, Synthesis and Characterization of the Peptides

We previously designed the β-sheet-forming nonapeptides (Ac-AIAKAXKIA-NH_2_) RU006 (X = L-2-naphthylalanine, NaI) [18,19,20] and (Ant^6^)-RU006 (X = L-2-anthrylalanine, Ant) [18,19] to form amphiphilic configurations when the peptides formed β-sheet structures. Two hydrophobic amino acids (isoleucine; Ile) at the 2nd and 8th positions and an aromatic amino acid (X) at the 6th position were placed on one side to form a hydrophobic face. Four less hydrophobic amino acids (alanine; Ala) at the 1st, 3rd, 5th and 9th positions and a hydrophilic lysine (Lys) at the 7th position were placed on the other (hydrophilic) side (Figure 1A). A Lys residue at the 4th position was placed near the aromatic amino acid at the hydrophobic face to interact with anionic AuCl_4_^−^ and PtCl_6_^2−^ to enrich and reduce the metal ions concentrated in peptide self-assemblies. Additionally, we designed the new nonapeptide (NaI^2^)-RU006, where Ile at the 2nd position was replaced with a NaI to provide two NaI residues (potential to provide four electrons per molecule), the same as (Ant^6^)-RU006 but with the same oxidation potential as RU006 for comparison (Figure 1A).

The peptides were synthesized using a standard solid-phase peptide synthesis method with Fmoc chemistry [22]. The crude peptides were purified by HPLC (Figure S1) and characterized by MALDI-TOFMS (Figure S2). In our previous study, ATR-FTIR measurements revealed that RU006 and (Ant^6^)-RU006 form antiparallel β-sheet structures [18]. We examined the secondary structure of newly synthesized (NaI^2^)-RU006 and observed two FTIR absorption bands around 1630 and 1680 cm^−1^ in the amide I region, originating from amide carbonyl stretching frequencies (1600–1700 cm^−1^) (Figure S3). These bands correspond to an antiparallel β-sheet structure, but a totally broad profile was observed in the amide I region, probably suggesting that (NaI^2^)-RU006 coexists in β-sheet and random structures due to steric hindrance caused by the two bulky naphthalene rings [23,24].

### 3.2. Reduction of Metal Ions by the Peptides

**Figure 2:**
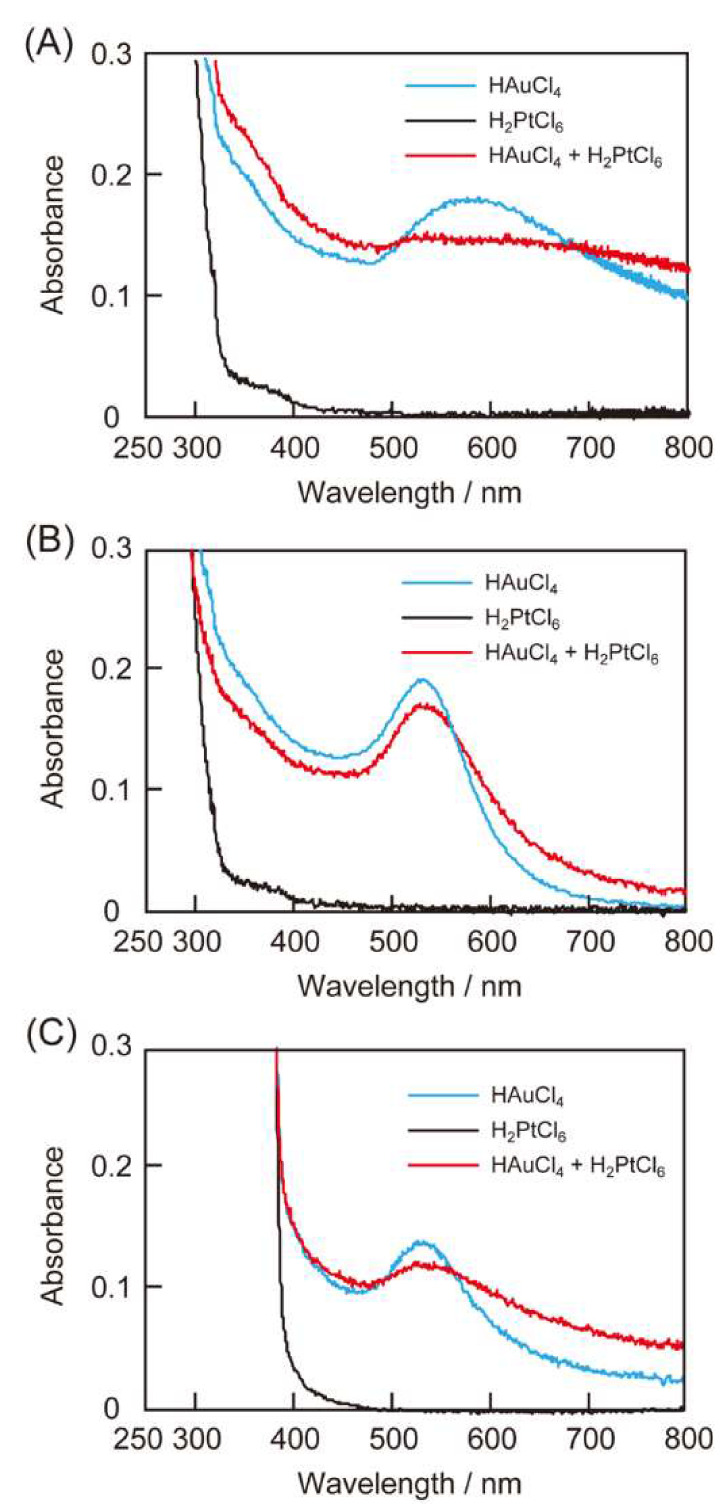
UV-Vis spectra of reaction mixtures of peptides (**A**) RU006, (**B**) (NaI^2^)-RU006 and (**C**) (Ant^6^)-RU006) and metal ion(s) (blue lines: HAuCl_4_ only, black lines: H_2_PtCl_6_ only and red lines: HAuCl_4_ + H_2_PtCl_6_). (Peptide) = 2.0 × 10^−4^ M and (HAuCl_4_) = (H_2_PtCl_6_) = 5.0 × 10^−5^ M in water. Each reaction was conducted at 40 °C for 24 h in the dark.

First, we examined the reducing activities of the peptides towards metal ions by preparing equivolume mixtures of aqueous solutions of peptide (4.0 × 10^−4^ M) and HAuCl_4_ (or H_2_PtCl_6_ or a mixture of HAuCl_4_ and H_2_PtCl_6_, each 1.0 × 10^−4^ M). Figure 2 shows the UV-Vis spectra of the peptide and metal ion reaction mixtures. A somewhat broad absorption peak was observed at 580 nm (Figure 2A), suggesting ribbon-like gold nanocrystal formation due to the reduction of HAuCl_4_ with RU006, as observed previously [18]. The reaction mixture comprising RU006, HAuCl_4_ and H_2_PtCl_6_ also afforded a broad absorption band spanning from 500 to 800 nm. However, the absorption spectrum of RU006 did not show any change in the presence of H_2_PtCl_6_. Figure 2B shows the reducing activities of (NaI^2^)-RU006 for noble metal ions. A sharp absorption band was observed at 530 nm for the reaction mixture comprising (NaI^2^)-RU006 and HAuCl_4_, indicating relatively spherical gold nanocrystal formations [25,26]. Similarly, the reaction mixture of (NaI^2^)-RU006, HAuCl_4_ and H_2_PtCl_6_ exhibited an absorption band at 530 nm, although it was slightly broadened. However, there was no large change in the absorption spectrum of (NaI^2^)-RU006 in the presence of H_2_PtCl_6_. Figure 2C shows the reducing activities of (Ant^6^)-RU006 for noble metal ions. The reaction mixture comprising (Ant^6^)-RU006 and HAuCl_4_ gave a sharp absorption band at 530 nm, as seen in Figure 2B, whereas that of (Ant^6^)-RU006, HAuCl_4_ and H_2_PtCl_6_ exhibited a broader absorption band. However, the absorption band of (Ant^6^)-RU006 did not change in the presence of H_2_PtCl_6_. These results suggest that a naphthalene ring or an anthracene ring in the peptides did not reduce H_2_PtCl_6_ but did reduce HAuCl_4_, which is potentially conducive for selective gold recovery from a mixture of HAuCl_4_ and H_2_PtCl_6_ through the selective reduction of HAuCl_4_. Differences in the absorption spectra of the reaction mixtures containing HAuCl_4_ and a mixture of HAuCl_4_ and H_2_PtCl_6_ could be due to the presence of platinum in addition to gold.

### 3.3. Characterization of Precipitates from Peptide/Noble Metal Reaction Mixtures

**Figure 3:**
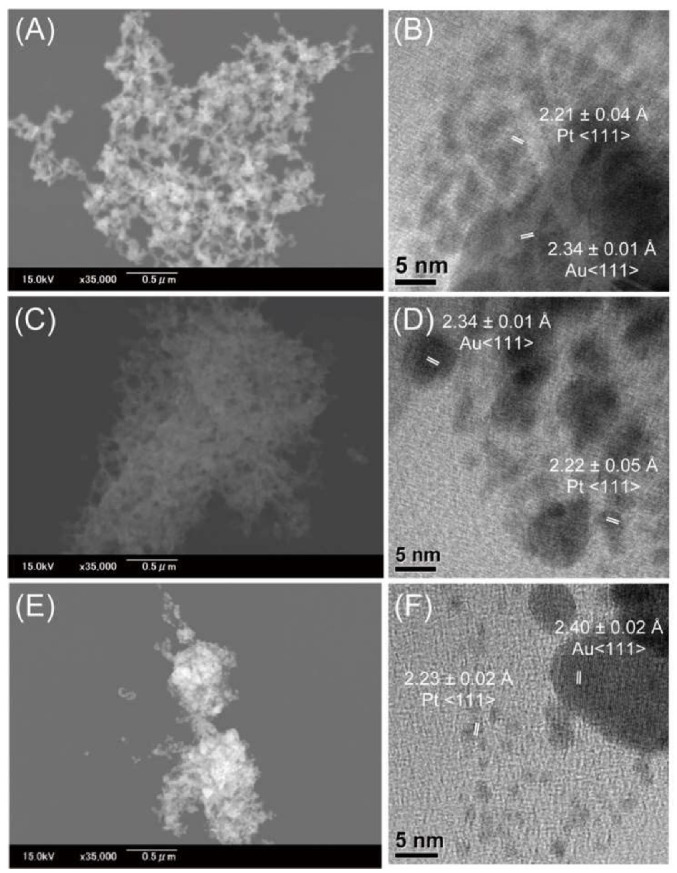
Field emission-scanning electron microscopy (FE-SEM) (**A**,**C**,**E**) and transmission electron microscopy (TEM) (**B**,**D**,**F**) images of precipitates from reaction mixtures of peptides ((**A**,**B**) RU006, (**C**,**D**) (NaI^2^)-RU006 and (**E**,**F**) (Ant^6^)-RU006) and metal ions (HAuCl_4_ and H_2_PtCl_6_). (Peptide) = 2.0 × 10^−4^ M and (HAuCl_4_) = (H_2_PtCl_6_) = 5.0 × 10^−5^ M in water. Each reaction was conducted at 40 °C for 24 h in the dark.

We characterized the products from reaction mixtures comprising the peptides, HAuCl_4_ and H_2_PtCl_6_ by collecting the reaction mix precipitates by centrifugation and analyzing their morphologies by FE-SEM and TEM. Figure 3A shows an FE-SEM image of the precipitate from a reaction mixture of RU006, HAuCl_4_ and H_2_PtCl_6_. Many ribbon-like nanostructures were observed, as seen in our previous study [18]. Figure 3B is a TEM image of the edge of a ribbon-like nanostructure such as that observed in Figure 3A and shows relatively large solid particles with a *d*-spacing of 2.34 Å corresponding to the Au<111> direction of elongation for face-centered cubic (fcc) crystals and small crystals with a *d*-spacing of 2.21 Å corresponding to the Pt<111> direction of elongation for the fcc crystal structure [27]. Figure 3C shows an FE-SEM image of many spherical nanostructures in the precipitate from a reaction mixture comprising (NaI^2^)-RU006, HAuCl_4_ and H_2_PtCl_6_. Figure 3D is a TEM image of the edge of such a spherical nanostructure and shows relatively large solid particles with a *d*-spacing of 2.34 Å corresponding to the Au<111> direction of elongation and small crystals with a *d*-spacing of 2.22 Å corresponding to the Pt<111> direction of elongation. Figure 3E shows an FE-SEM image of mixed nanostructures of spheres and short wires in the precipitate from a reaction mixture comprising (Ant^6^)-RU006, HAuCl_4_ and H_2_PtCl_6_. Figure 3F is a TEM image of the edge of such a nanostructure showing relatively large solid particles with a *d*-spacing of 2.40 Å corresponding to the Au<111> direction of elongation and small crystals with a *d*-spacing of 2.23 Å corresponding to the Pt<111> direction of elongation. These results suggest that the precipitates from reaction mixtures comprising the peptides, HAuCl_4_ and H_2_PtCl_6_ are metallic, with metallic gold forming relatively large particles and metallic platinum forming small particles.

### 3.4. Selectivity of the Peptide-Based Recovery System for Gold

**Figure 4:**
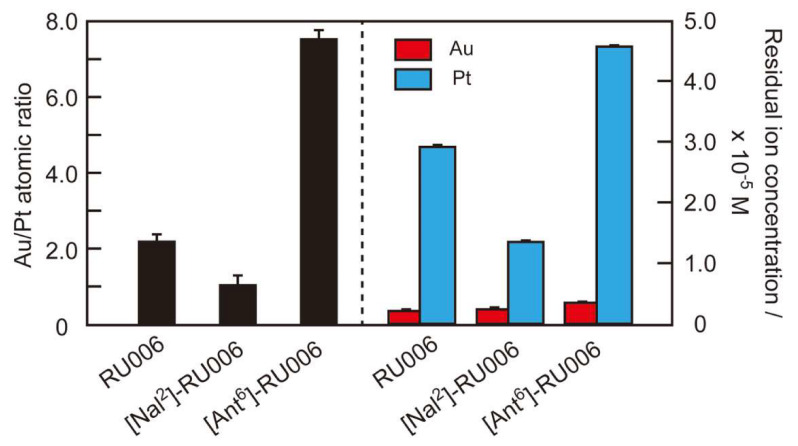
Gold/platinum (Au/Pt) atomic ratios of the metallic precipitates determined by energy-dispersive X-ray spectroscopy-scanning electron microscopy (EDS-SEM) (left axis) and residual metal ion concentrations in the supernatants determined by inductively coupled plasma-optical emission spectroscopy (ICP-OES) (right axis) with the peptides RU006, (NaI^2^)-RU006 and (Ant^6^)-RU006). Samples were separated by centrifuging the reaction mixtures of peptides and metal ions (HAuCl_4_ + H_2_PtCl_6_). (Peptide) = 2.0 × 10^−4^ M and (HAuCl_4_) = (H_2_PtCl_6_) = 5.0 × 10^−5^ M in water. Each reaction was conducted at 40 °C for 24 h in the dark (mean ± SD, *n* = 3).

To understand the selectivity of the peptide-based recovery system for gold, we used EDS-SEM (Figure 4, left axis) and ICP-OES (Figure 4, right axis) to determine the compositions of the metallic precipitates and supernatants provided by centrifugation of the reaction mixtures comprising the peptides, HAuCl_4_ and H_2_PtCl_6_. The Au/Pt atomic ratios for the precipitates for RU006 and (NaI^2^)-RU006 were 2.2 ± 0.2 and 1.1 ± 0.2, respectively, indicating that RU006 solidified HAuCl_4_ twice as efficiently as H_2_PtCl_6_. In contrast, (NaI^2^)-RU006 showed poor gold selectivity. Surprisingly, (Ant^6^)-RU006 mineralized noble metal ions with an Au/Pt atomic ratio of 7.5 ± 0.2, suggesting that an anthracene ring is a more suitable reducing agent than a naphthalene ring for selective gold reductions from the reaction mixtures.

ICP-OES analysis of the supernatants of the reaction mixtures containing RU006 revealed a residual metal ion concentration of 2.5 × 10^−6^ M (95% recovery) for Au(III)/Au(I) and 2.89 × 10^−5^ M (44% recovery) for Pt(IV)/Pt(II). ICP-OES analysis of reaction mixtures containing (NaI^2^)-RU006 gave residual ion concentrations of 2.4 × 10^−6^ M (95% recovery) for Au(III)/Au(I) and 1.35 × 10^−5^ M (74% recovery) for Pt(IV)/Pt(II). The recoveries obtained using (Ant^6^)-RU006 were 3.5 × 10^−6^ M (93% recovery) and 4.54 × 10^−5^ M (12% recovery) for Au(III)/Au(I) and Pt(IV)/Pt(II), respectively. These recovery ratios (Au recovery %/Pt recovery %) determined by ICP-OES are in good agreement with the Au/Pt ratios by EDS-SEM. These quite high recovery yields for gold suggest that gold ions were enriched by peptide self-assembly, reduced to the metallic state and densified, enabling the easy separation of the metallic solids by simple centrifugation. However, the residual Pt ion concentration was strongly dependent on the aromatic side chains in the peptides, with (Ant^6^)-RU006 providing the most selective gold recovery of the peptides examined here.

### 3.5. Mechanism of Selective Gold Recovery by the Peptide-Based System

**Figure 5:**
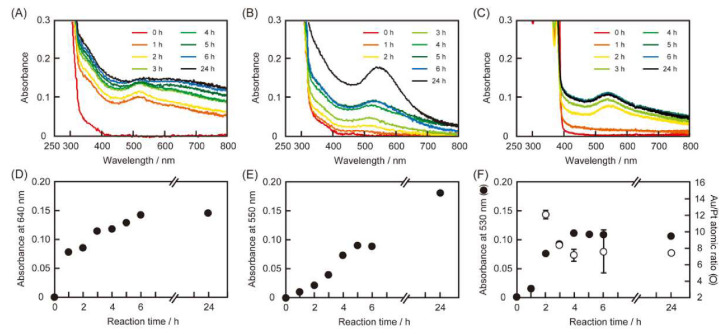
UV-Vis spectra of the reaction mixtures of peptides (**A**,**D**) RU006, (**B**,**E**) (NaI^2^)-RU006 and (**C**,**F**) (Ant^6^)-RU006 and metal ions (HAuCl_4_ + H_2_PtCl_6_) as a function of time. (Peptide) = 2.0 × 10^−4^ M and (HAuCl_4_) = (H_2_PtCl_6_) = 5.0 × 10^−5^ M in water. Each reaction was conducted at 40 °C in the dark. (**F**, right axis) T-ime-dependent Au/Pt atomic ratios of the precipitates determined by EDS-SEM. (mean ± SD, *n* = 3).

We examined changes in the UV-Vis spectra of the reaction mixtures containing the peptides, HAuCl_4_ and H_2_PtCl_6_ as a function of time to understand the mechanism of the peptide-based selective gold recovery. Figure 5A,D shows the time-dependent profiles of the reduction of metal ions by RU006. The initial increase in absorbance at 510 nm corresponds to a spherical gold nanoparticle formation. The subsequent increase with time of the absorbance at 640 nm corresponds to thr aggregation of the spherical gold nanoparticles up to 6 h. Figure 5B,E shows slow time-dependent spectral changes for (NaI^2^)-RU006, despite having two naphthalene rings, compared to one in RU006. The absorption band gradually red-shifted with time from 520 to 550 nm, suggesting an aggregation of the gold nanocrystals. In contrast, (Ant^6^)-RU006 synthesized metallic nanocrystals within 4 h, much quicker than the other peptides (Figure 5C,F, closed circles). We used EDS-SEM to observe the time-dependency of the Au/Pt atomic ratio for the metallic precipitate obtained from a mixture of (Ant^6^)-RU006, HAuCl_4_ and H_2_PtCl_6_ (Figure 5F, open circles). The Au/Pt atomic ratio for the metallic precipitate was about 12.3 after 2 h from the start of the reaction, then decreased to 7.5 by 4 h and remained essentially constant. These results suggest that gold nanocrystals first formed by the reduction of HAuCl_4_ by (Ant^6^)-RU006 at an early stage, and then, platinum nanocrystals were slowly generated until the reaction was complete, resulting in a high Au/Pt atomic ratio [28,29]. Such rapid crystallization of Au(III) by (Ant^6^)-RU006 might limit the formation of the stable Au(I) intermediate that provides electrons to Pt(IV)/Pt(II) for the formation of platinum precipitates. The importance of a limited Au(I) formation might be supported by our finding that the peptides do not reduce H_2_PtCl_6_ in the absence of HAuCl_4_ (Figure 2) and that the change in the Au/Pt ratio for the (Ant^6^)-RU006 system terminated when the reaction ended (Figure 5F, open circles). The two bulky naphthalene rings in (NaI^2^)-RU006 inhibit (NaI^2^)-RU006 self-association, and thus, the concentration of intermediate Au(I) remained sufficiently high to provide electrons to Pt(IV)/Pt(II), resulting in poor gold selectivity. From the viewpoint of kinetics, the rapid reduction of HAuCl_4_ could thus be quite important for gold selectivity by the present peptide-based recovery process from a homogenous aqueous solution containing relatively low concentrations of HAuCl_4_ and H_2_PtCl_6_.

Meanwhile, from the viewpoint of structural characteristics of the peptides, unfortunately, it is yet unclear which amino acids play important roles for such gold selectivity in the recovery process. More detailed mechanisms would request the specification of amino acids essential for the selectivity by deleting amino acids comprising (Ant^6^)-RU006 from its N and/or C termini one-by-one and the rearrangement of Ant and Lys residues to keep the distance between an aromatic ring and a cationic side chain. We are now working on it, and the results will be published elsewhere.

### 3.6. Role of Peptide Self-Assembly for Selective Gold Recovery

**Figure 6:**
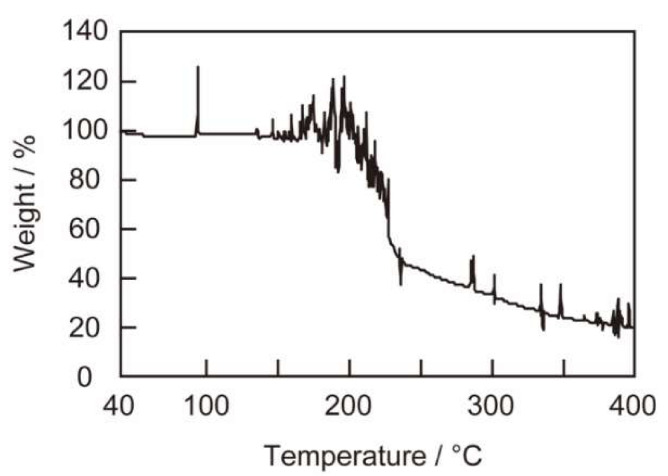
Thermogravimetric profile of the precipitate generated by a reaction mixture comprising (Ant^6^)-RU006 and metal ions (HAuCl_4_ + H_2_PtCl_6_). y-Axis shows weight changes of the precipitates relative to the weight at 40 °C as a function of temperature. The rate of the temperature increase was 5 K min^−1^.

We examined the compositions of the precipitates obtained from reaction mixtures comprising (Ant^6^)-RU006, HAuCl_4_ and H_2_PtCl_6_ to understand the role of peptide self-assembly in peptide-based selective gold recovery. Figure 6 shows the results of a thermal gravimetric analysis of metallic precipitates (0.365 mg) obtained by simple centrifugation. The temperature was increased from 40 °C to 400 °C. The weight of the precipitate drastically decreased at 230 °C to 19% (0.069 mg, 3.5 × 10^−7^ mol, average atomic weight = 196.8 calculated from the Au/Pt atomic ratio of 7.5 in Figure 4, left axis) of the weight prior to heating. This decrease is likely due to the decomposition of the peptides (0.296 mg, 2.8 × 10^−7^ mol) coprecipitated with the metallic solids. The metallic content of the precipitates was roughly calculated to be (Ant^6^)-RU006/metal = 4/5 (mol/mol). Thus, about 20% of the peptides in the reaction mixture enriched and reduced metal ions and densified the products for easy separation during peptide self-assembly.

### 3.7. Gold Recovery Using Common Reducing Agents

**Figure 7:**
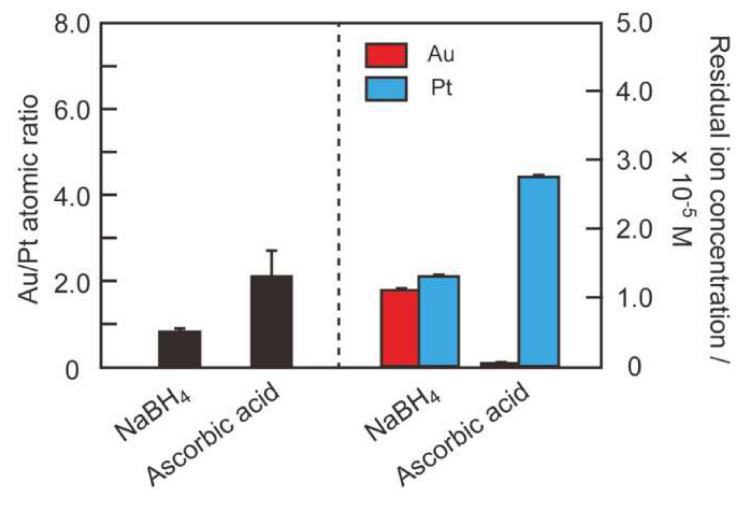
Au/Pt atomic ratios of the metallic precipitates determined by EDS-SEM (left axis) and residual metal ion concentrations in the supernatants determined by ICP-OES (right axis) with the common reducing agents NaBH_4_ and ascorbic acid. Samples were separated by centrifuging reaction mixtures of peptides and metal ions (HAuCl_4_ + H_2_PtCl_6_). (Reducing agent) = 1.0 × 10^−3^ M and (HAuCl_4_) = (H_2_PtCl_6_) = 5.0 × 10^−5^ M in water. The reactions were conducted at 40 °C for 24 h in the dark (mean ± SD, *n* = 3).

Lastly, we compared the selectivities of the common reducing agents NaBH_4_ and ascorbic acid (each 1.0 × 10^−3^ M) for gold recovery from a mixture of HAuCl_4_ and H_2_PtCl_6_ (each 5.0 × 10^−5^ M) (Figure 7). Au/Pt atomic ratios of the precipitates determined by EDS-SEM analysis were 0.9 ± 0.1 and 2.1 ± 0.6 for NaBH_4_ and ascorbic acid, respectively. Residual ion concentrations determined by ICP-OES were 1.1 × 10^−5^ M (78% recovery for Au(III)/Au(I)) and 1.3 × 10^−5^ M (74% recovery for Pt(IV)/Pt(II)) for NaBH_4_ and 5.0 × 10^−7^ M (99% recovery for Au(III)/Au(I)) and 2.8 × 10^−5^ M (46% recovery for Pt(IV)/Pt(II)) for ascorbic acid. These results suggest that NaBH_4_, a strong reducing agent, equally reduced HAuCl_4_ and H_2_PtCl_6_, whereas ascorbic acid was more selective but much less so than (Ant^6^)-RU006 under the same conditions, suggesting that the use of reducing agents with suitable oxidation potentials is important for gold selectivity.

## 4. Conclusions

In this study, we investigated selective gold recovery by RU006, (NaI^2^)-RU006 or (Ant^6^)-RU006 (each 2.0 × 10^−4^ M) from a homogenous aqueous solution comprising a mixture of dilute HAuCl_4_ and H_2_PtCl_6_ (each 5.0 × 10^−5^ M). Precipitates from the reaction mixtures were obtained by simple centrifugation and resulted from peptide self-assembly enrichment and the subsequent reduction of the noble metal ions to form dense products. The (Ant^6^)-RU006 contains an anthracene ring and showed high gold selectivity (Au/Pt atomic ratio = 7.5), and the reduction of H_2_PtCl_6_ was significantly inhibited relative to the other peptide-based systems. Common reducing agents such as NaBH_4_ and ascorbic acid reduced a mixture of HAuCl_4_ and H_2_PtCl_6_, but their gold selectivity was low compared with the (Ant^6^)-RU006 system, probably because the oxidation potentials must be suitable for the reduction of gold ions. Rapid reactions involving peptide-noble metal ion interactions, enrichments and the reductions of noble metal ions and the densification of the products during peptide self-assembly are important for high gold selectivity in noble metal recovery processes by peptide-based systems. We here demonstrated a peptide-based selective gold recovery method that meets the following criteria for a practical system: (i) high metal element selectivity, (ii) one-pot operation, (iii) easy separation and (iv) a monophasic reaction in (homogenous) aqueous solution under dilute conditions. We anticipate that this system will be applicable to the sequential separation of several noble metal ions such as Au, Pd, Pt, Ir and Rh following the development of aromatic peptides suitable for each noble metal of interest. However, the preparations of peptides are costly and need a lot of reagents and solvents. Therefore, downsizing such active peptides by deleting amino acids from their N and/or C termini one-by-one to specify the amino acids essential for selective noble metal recovery would be the next step. We are now working on downsizing (Ant^6^)-RU006 for the specification of amino acids essential for the expression of gold selectivity in the recovery process. We believe that the findings provided from such peptide-based experiments encourage us to redesign nonexpensive, nonpeptidyl molecules to be synthesized in an environmentally friendly manner for large-scale noble metal recovery processes.

## Supplementary Materials

The following are available online at https://www.mdpi.com/1422-0067/21/14/5060/s1: Figure S1: Analytical HPLC profile for [Nal^2^]-RU006, Figure S2: MALDI-TOFMS for [Nal^2^]-RU006, Figure S3: ATR-FTIR spectra of [Nal^2^]-RU006.
