# Supplementary material for: Selective Gold Recovery from Homogenous Aqueous Solutions Containing Gold and Platinum Ions by Aromatic Amino Acid-Containing Peptides
Source: Int J Mol Sci. 2020 Jul 17;21(14):5060. doi: 10.3390/ijms21145060 (PMC7403969; doi:10.3390/ijms21145060)
Supplement: Supplementary file 1 [file ijms-21-05060-s001.pdf]

Supplementary Material

1. Analytical HPLC profile for [Nal^2]-RU006.

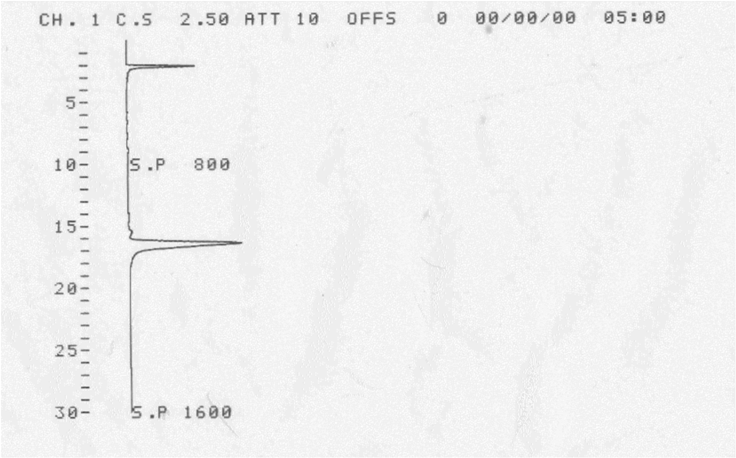

Figure S1. Analytical HPLC profile for [Nal^2]-RU006. Conditions: acetonitrile content: 32% (t = 0 min) 40% (t = 30 min); UV Detection: 220 nm.

2. MALDI-TOF-MS for [Nal^2]-RU006.

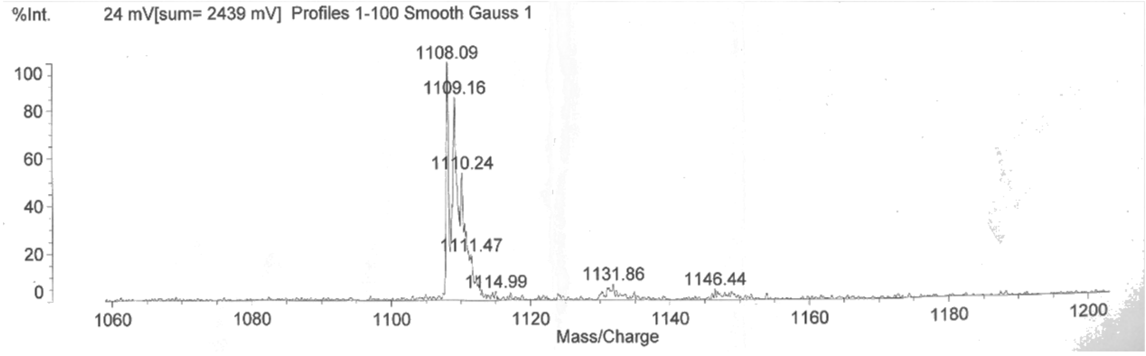

Figure S2. MALDI-TOF-MS for [Nal^2]-RU006. Calculated molecular weight: 1108.37 observed molecular weight 1108.09 $[M + H]^+$, 10131.86 $[M + Na]^+$, 1146.44 $[M + K]^+$.

3. ATR-FTIR for [Nal^2]-RU006.

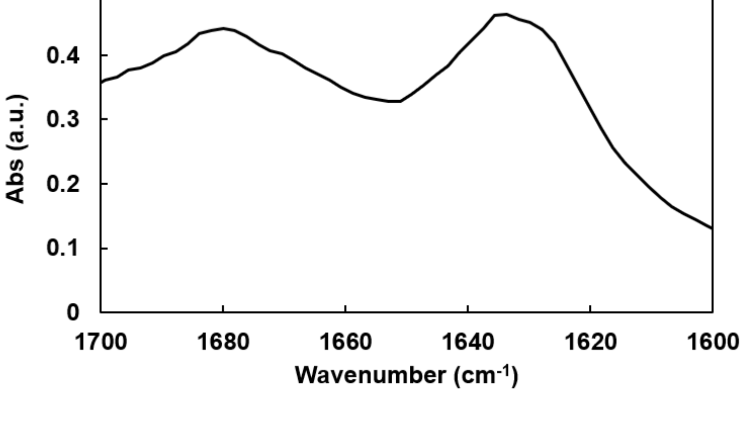

Figure S3. ATR-FTIR spectra of peptide films prepared from aqueous solution of [Nal^2]-RU006 ([peptide] = 1.0 mM in water for 1 day).
